# Diagnostic performance of elastosonography in the differential diagnosis of benign and malignant salivary gland tumors: A meta-analysis

**DOI:** 10.3389/fonc.2022.954751

**Published:** 2022-09-20

**Authors:** Jiangfeng Wu, Zhijuan Zhou, Xiaoyun Wang, Yun Jin, Zhengping Wang, Guilong Jin

**Affiliations:** ^1^ Department of Ultrasound, Dongyang Hospital of Wenzhou Medical University, Dongyang, China; ^2^ Department of Ultrasound, Tianxiang East Hospital, Yiwu, China; ^3^ Department of Nephrology, Dongyang Hospital of Wenzhou Medical University, Dongyang, China; ^4^ Department of Ultrasound, Dongyang People’s Hospital, Dongyang, China

**Keywords:** elastosonography, conventional ultrasound, diagnosis, salivary gland tumor, meta-analysis

## Abstract

**Purpose:**

The clinical practice of elastosonography for the detection of salivary gland tumors is still a controversial issue. The objective of this meta-analysis was to evaluate the effect of elastosonography for the diagnosis of salivary gland tumors and to compare the diagnostic value of elastosonography and conventional ultrasound in the diagnosis of salivary gland tumors.

**Methods:**

A comprehensive literature search through PubMed, EMBASE, and Cochrane Library was carried out from inception to November 2021. Two researchers independently extracted the data from the enrolled papers using a standard data extraction form. The pooled sensitivity, specificity, positive likelihood ratio (PLR), negative likelihood ratio (NLR), diagnostic odds ratio (DOR), and area under the curve (AUC) were calculated to evaluate the diagnostic performance of elastosonography. The Quality Assessment of Diagnostic Accuracy Studies—2 (QUADAS-2) tool was utilized to evaluate the quality of each included study. Meta-DiSc version 1.4, Review Manager 5.3, and StataSE 15 were used.

**Results:**

Sixteen studies with a total of 1105 patients with 1146 lesions were included in this meta-analysis. The pooled sensitivity, specificity, PLR, NLR, and DOR of elastosonography for the differentiation between benign and malignant salivary gland tumors were 0.73 (95%CI, 0.66–0.78), 0.64 (95%CI, 0.61–0.67), 2.83 (95%CI, 1.97–4.07), 0.45 (95%CI, 0.32–0.62), and 9.86 (95%CI, 4.49–21.62), respectively, with an AUC of 0.82. Four studies provided data regarding the conventional ultrasound for the differentiation between benign and malignant salivary gland tumors. The pooled sensitivity, specificity, and DOR were 0.62 (95%CI, 0.50–0.73), 0.93 (95%CI, 0.90–0.96), and 25.07 (95%CI, 4.28–146.65), respectively. The meta-regression and subgroup analyses found that assessment methods were associated with significant heterogeneity, and quantitative or semiquantitative elastosonography performed better than the qualitative one.

**Conclusions:**

Elastosonography showed a limited value for diagnosing malignant salivary gland tumors; it could be considered as a supplementary diagnostic technology to conventional ultrasound, and quantitative or semiquantitative elastosonography was superior to the qualitative one.

## Introduction

Salivary gland tumors are rare, which account for 2%–6% of all head and neck tumors, with an annual incidence ranging from one to five cases per 100,000 population. The most common benign tumors of the salivary glands include pleomorphic adenoma (PA) and Warthin tumor (WT), and 85% of the tumors arise in the parotid gland (1, 2). The treatment strategy of salivary gland tumors depends primarily on its pathology, and preoperative diagnosis of the tumor entity directly affects the selection of surgical procedure; therefore, to determine whether a tumor is benign or malignant is crucial (3–5).

Currently, it is not an easy task to accurately identify benign from malignant salivary tumors because of a broad variety of potential differential diagnoses and the lack of specific imaging characteristics (6–8). Conventional ultrasound (US) is the first-line imaging technique for the diagnosis of the salivary gland tumors as it is a widely available, noninvasive, nonradioactive, and cost-effective method (6). However, the diagnostic accuracy of conventional US depends on the sonographer’s diagnostic skill and experience, and there are overlaps of sonographic appearances among different pathological tumors (6, 7). Consequently, the accuracy of conventional US for salivary tumors is less than satisfactory (6–8). Magnetic resonance imaging (MRI) and computed tomography (CT) are also the primary imaging modalities for evaluating salivary gland tumors (9–11). While they can find tumors with high sensitivity, these are less accurate for predicting histology due to an appreciable overlap of imaging findings between different pathological types of salivary gland tumors (8, 11). Thus, acquiring the histopathology of tumors by US-guided fine-needle aspiration cytology (FNAC) or core-needle biopsy (CNB) continues to be necessary before the surgical procedure (12). However, these techniques are invasive and could possibly lead to some complications such as pain and hemorrhage. Thus, an alternative imaging technique providing additional information for identifying salivary gland tumors would be greatly valuable.

Elasticity is an important feature revealing tissue stiffness, which is defined as the rate of change of spatial displacement due to the tensile stress on the tissue under applied pressure (13). Elastosonography is a simple approach that determines tissue stiffness as qualitative, semiquantitative, or quantitative, which has been demonstrated to be useful for the evaluation of thyroid nodules, breast tumors, and cirrhosis (14–17).

The clinical practice of elastosonography for the detection of salivary gland tumors is still a controversial issue, as the diagnostic performance is variable in different studies, with the sensitivity ranging from 38% to 100% and specificity from 26% to 97% (18–21). Thus, we thought it is necessary and timely to summarize currently available data to provide valuable information for clinical practice. The objective of this meta-analysis was to evaluate the effect of elastosonography for the diagnosis of salivary gland tumors and to compare the diagnostic value of elastosonography and conventional US in the diagnosis of salivary gland tumors.

## Materials and methods

This meta-analysis was performed in accordance with the Preferred Reporting Items for Systematic Reviews and Meta-analysis (PRISMA) Statement (22).

### Literature search

A comprehensive literature search through PubMed, EMBASE, and Cochrane Library was carried out from inception to November 2021 to identify English-language studies on elastosonography for diagnosing salivary gland tumors. The search strategy was in accordance with the combination of the medical subject heading (MeSH) terms, key words, and word variants for “elastosonography”, “elastography”, “ultrasound elastography”, “ultrasonic elastography”, “parotid gland tumor”, and “salivary gland tumor”. Reference lists of the included papers were also manually screened to detect additional relevant studies. Details of the strategy of searching are provided in **Supplementary Table 1**.

### Inclusion and exclusion criteria

Two researchers independently scanned the titles and abstracts of the relevant papers. The inclusion and exclusion criteria were defined to increase reproducibility and validity before identifying the studies. All the disagreements were resolved by consensus. All potentially relevant articles satisfying the following criteria were included: (1) diagnostic studies were included; (2) studies assessing the diagnostic performance of elastosonography in differentiating benign from malignant salivary gland tumors were included; and (3) reference standards such as postoperative pathology and/or biopsy results were adopted. The exclusion criteria for the studies were as follows: (1) case reports, reviews, consensus statements, editorial comments, letters, conference reports, and unpublished articles were excluded; (2) studies without sufficient data to construct a 2 × 2 contingency table were excluded; and (3) studies that were not published in English were excluded.

### Data extraction and processing

Two researchers independently extracted the data from the enrolled papers using a standard data extraction form. All the disagreements were resolved by consensus. For included studies, the following items were extracted: author, year of publication, country, study type, sample method, blinding method, sex, number of lesions, age, mean size of tumors, site of lesions, technology, index of elastography, threshold value, reference standard, ultrasound equipment and probe, sensitivity, and specificity.

### Quality assessment

The Quality Assessment of Diagnostic Accuracy Studies—2 (QUADAS-2) tool recommended by the Cochrane collaboration was utilized to evaluate the quality of each included study (23). The QUADAS-2 tool comprises two main categories, namely the risk of bias of four domains and the clinical applicability of three domains. The four domains include patient selection, index test, reference standard, and flow and timing. Every domain is assessed for risk of bias, and the first three domains are assessed for clinical applicability. The quality assessment was performed using the RevMan 5.3 software (Nordic Cochrane Centre, Copenhagen, Denmark).

### Statistical analysis

From the enrolled papers, a bivariate effect model was utilized in this study to calculate the pooled sensitivity, specificity, positive likelihood ratio (PLR), negative likelihood ratio (NLR), and diagnostic odds ratio (DOR) with corresponding 95% confidence intervals (CIs), which revealed the diagnostic performance of elastosonography in differentiating benign from malignant salivary gland tumors. The presence of a threshold effect was determined by analyzing the Spearman correlation coefficient between sensitivity and the false-positive rate, through a p < 0.05 indicating threshold effect. In addition, the summary receiver operator curve (SROC) was developed, and this allowed us to compute the area under the curve (AUC). The AUC values of 0.5–0.7, 0.7–0.9, and >0.9 indicate low, moderate, and perfect diagnostic performance, respectively (24). The Higgins *I* ^2^ statistic and Q test were utilized to evaluate the heterogeneity of the study with *I* ^2^ > 50% showing significant heterogeneity (25). A random-effects model is adopted when the significant heterogeneity is found across studies; otherwise, a fixed-effects model is adopted. The Deeks’ funnel plot was generated to evaluate publication bias (26) through a p < 0.05 indicating potential publication bias.

Meta-regression and subgroup analyses using several covariates were conducted to investigate the potential factors of heterogeneity: study design (prospective vs. others), year of publication (2010–2013 vs. 2014–2020), diagnostic measurement (quantitative or semiquantitative vs. qualitative), and blinding method (yes vs. unclear). All the above statistical analyses were carried out by Meta-DiSc version 1.4 and StataSE 15 (Stata Corporation, College Station, TX).

## Results

### Literature search

**Figure 1:**
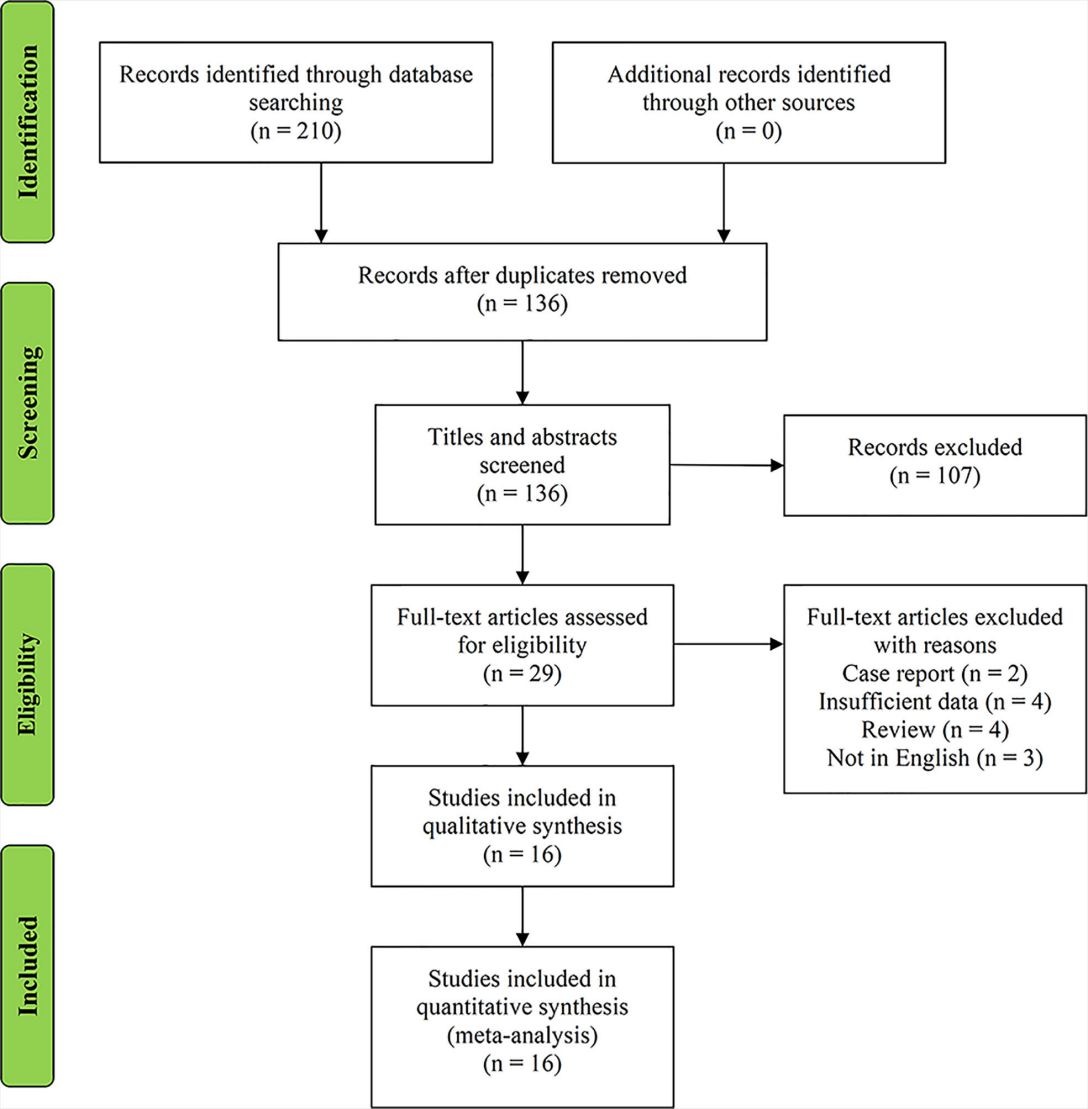
Flowchart of study selection.

On the basis of the predefined MeSH terms, key words, and word variants, our database search initially identified 210 papers for consideration. PubMed found 95 studies, EMBASE identified 88, and the Cochrane Library discovered 27. After excluding the duplications, the remaining 136 potentially eligible original papers were further reviewed. Furthermore, according to the inclusion criteria in the study selection process, 107 studies were discarded after screening the titles and abstracts. Twenty-nine papers were assessed by reviewing the full text, of which 13 were further excluded. Finally, 16 studies were included in this meta-analysis. **Figure 1** shows the detailed flowchart of the literature search.

### Characteristics of included studies

The 16 included studies were published from 2010 to 2020 and written in English (18–21, 27–38). A total of 1105 patients with 1146 lesions were included in these studies. Strain elasticity (SE) was used in 11 studies; shear wave velocity (SWE) was used in one study; acoustic radiation force impulse (ARFI) was used in three studies; and SE and ARFI were used in one study. Ten studies included parotid tumors only, while other studies included parotid, submandibular, or sublingual tumors. Quantitative or semiquantitative methods were utilized in four studies, while qualitative assessment methods were used in 12 studies. More detailed data extracted from the enrolled studies are available in **Tables 1** and **2**. The histopathological results of the included studies are revealed in **Supplementary Table 2**.

**Table 1:** Primary data extracted from the included studies for meta-analysis.

Author	Year	Country	Study type	Sample method	Blinding method	Number of lesions	Male/female	Age, year (mean or range)	Mean size of tumors, mm	Site of lesions
Bhatia et al. (27)	2010	China	NR	Consecutive	Single blind	65	48/13	60.5	26	Parotid (57) and submandibular (8)
Dumitriu et al. (28)	2011	Romania	NR	Consecutive	Single blind	74	37/29	50.8	29.54	Parotid (63) and submandibular (11)
Klintworth et al. (18)	2012	Germany	R	NR	Single blind	57	27/30	53.3	NR	Parotid
Yerli et al. (29)	2012	Turkey	P	Consecutive	Single blind	36	NR	NR	19.5	Parotid (30) and submandibular (6)
Celebi et al. (30)	2012	Turkey	P	Consecutive	Single blind	81	36/39	Men: 44.75; women: 49.44	NR	Parotid
Badea et al. (19)	2013	Romania	P	NR	NR	20	15/5	40-72	NR	Parotid
Wierzbicka et al. (21)	2013	Poland	P	Consecutive	NR	43	16/27	54	NR	Parotid
Yu et al. (31)	2016	China	NR	NR	Double blind	51	NR	45	NR	Parotid
Zhou et al. (32)	2016	China	R	Consecutive	Single blind	40	26/14	44	24.9	Parotid (29) and submandibular (11)
Cortcu et al. (33)	2017	Turkey	P	Consecutive	Single blind	39	22/17	52	PA: 27.8; WT: 33; other benign: 28; malignant: 25.6	parotid
Mansour et al. (20)	2017	Germany	P	NR	NR	202	NR	58.6	NR	Parotid
Cantisani et al. (34)	2017	Italy	P	Consecutive	Single blind	63	36/29	56	NR	Parotid
Altinbas et al. (35)	2017	Turkey	P	Consecutive	NR	54	26/20	60.01	23.68	Parotid
Liu et al. (36)	2018	China	P	Consecutive	Single blind	76	40/36	47.24	Benign: 24.53; malignant: 25.05	Parotid
Karaman et al. (37)	2019	Turkey	P	NR	Single blind	60	30/30	48.8	24.36	Parotid (42) and submandibular (18)
Matsuda et al. (38)	2020	Japan	R	Consecutive	Single blind	185	103/65	Benign: 62.8; malignant: 62.7	Benign: 27.6; malignant: 31.5	Parotid (169), submandibular (15), and sublingual (1)

NR, not reported; P, prospective; R, retrospective; PA, pleomorphic adenocarcinoma; WT, Warthin tumor.

**Table 2:** Characteristics of the included studies.

Author	Technology	Index of elastography	Threshold value	Reference standard	US equipment and probe	Sen (%)	Spe (%)
Bhatia et al. (27)	SE	4-point	≥ 3	Surgery or biopsy	Philips IU22 and Siemens Acuson Premium Edition; a 5- to 12-MHz linear probe and a 13.5-MHz linear probe	83	47
Dumitriu et al. (28)	SE	4-point	≥ 3	Surgery	EUB 8500, Hitachi; a 6- to 13-MHz linear probe	72	57
Klintworth et al. (18)	SE	Garland sign or not	Garland sign	Surgery	Acuson S2000; a 9-MHz linear probe	38	96
Yerli et al. (29)	SE	4-point	≥ 3	Surgery or biopsy	EUB-7000 ultrasound system; a 5- to 13-MHz linear probe	75	64
Celebi et al. (30)	SE	4-point	≥ 3	Surgery or biopsy	Siemens Acuson S2000 US; a 13-MHz probe	59	61
Badea et al. (19)	SE or ARFI	NR	NR	Surgery	GE 7, GE 8, GE 9, iU22 Phillips, and Siemens S 2000; a 7- to 11-MHz linear probe	100	50
Wierzbicka et al. (21)	SE	5-point	≥ 4	Surgery	AIXPLORER equipment; a Linear SL-15-4 transducer	40	97
Yu et al. (31)	SWE	SWV	2.76	Surgery	ACUSON S2000; a 7- to 12-MHz linear probe	69	97
Zhou et al. (32)	VTI (ARFI)	6-point	≥ 4	Surgery or biopsy	Siemens Acuson S2000; a 9L4 linear probe	63	81
Cortcu et al. (33)	SE	Strain ratio	2.1	Surgery or biopsy	Aplio XG SSA-790A; a 12-MHz linear probe	83	97
Mansour et al. (20)	SE	3-point	≥ 2	Surgery	Acuson S2000; a 9- to 14-MHz linear probe	69	26
Cantisani et al. (34)	SE	Elasticity contrast index	>3.5	Surgery or biopsy	ACCUVIX A30, RS 80 A; a 10- to 18-MHz linear probe	94	89
Altinbas et al. (35)	SE	0-6	3	Biopsy	Logiq S7 Expert machine; a 9L-D linear probe	70	66
Liu et al. (36)	VTQ (ARFI)	SWV	2.445 m/s	Surgery or biopsy	Siemens Acuson S2000; 14L5 linear probe and curvilinear probe	80	92
Karaman et al. (37)	SE	4-point	≥ 3	Histopathology	Acuson Antares; a 6- to 13-MHz linear probe	100	66
Matsuda et al. (38)	VTI (ARFI)	4-point	≥ 3	Surgery or biopsy	Siemens Acuson S2000; a 4- to 9-MHz or 14-MHz linear probe	77	64

SE, strain elasticity; ARFI, acoustic radiation force impulse; VTI, virtual touch imaging; VTQ, virtual touch quantification; NR, not reported; SWV, shear wave velocity; Sen, sensitivity; Spe, specificity.

### Quality assessment

**Figure 2:**
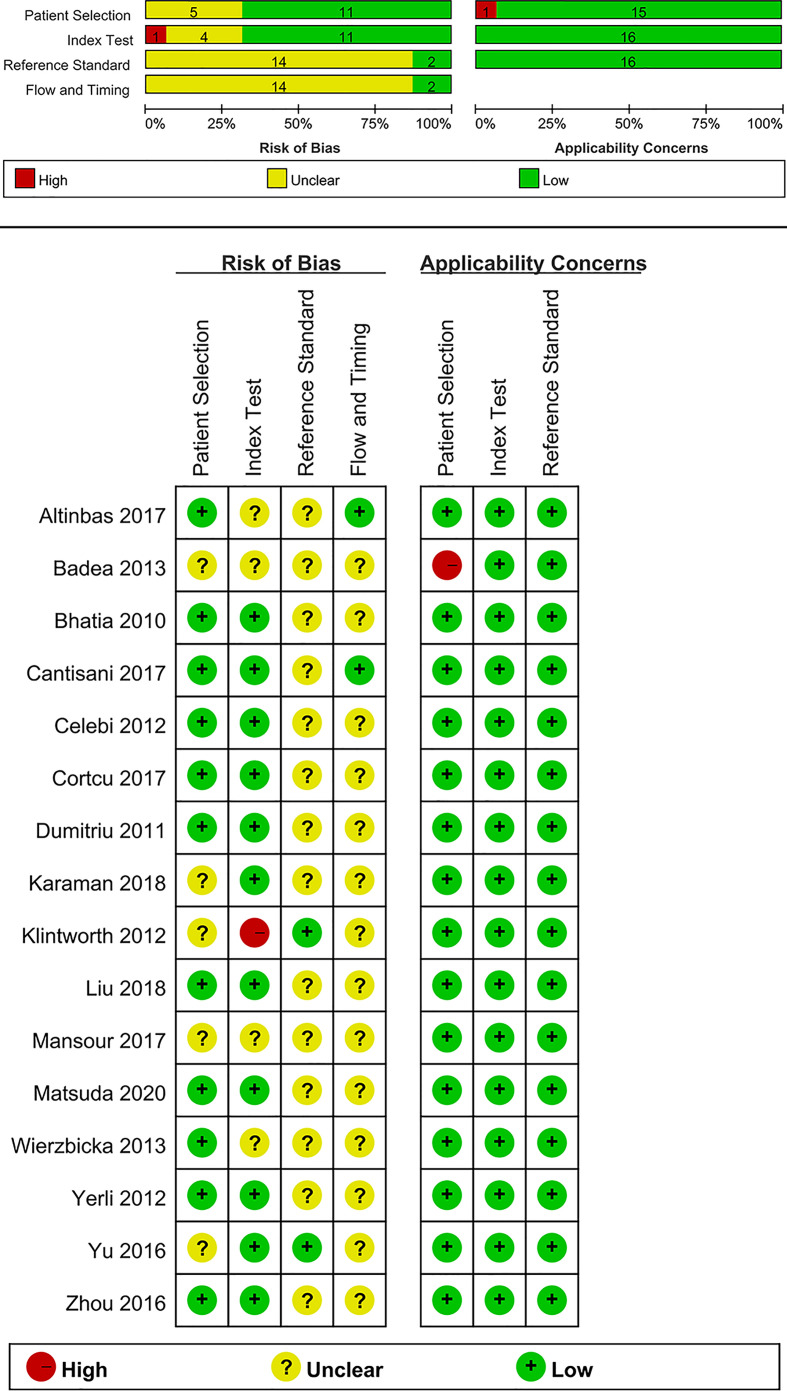
Summary of risk of bias and applicability concerns.

Quality assessment of each study based on the QUADAS-2 tool is shown graphically in **Figure 2**. Concerning the patient selection domain, five studies were thought to be “unknown” (18–20, 31, 37) because the sample method of patient selection was not definitely mentioned. Concerning the index test domain, four studies (19–21, 35) were thought to be “unknown” because the blinded status of the reference standard was not definitely mentioned; one study was considered as “high” because the sonographer was aware of the histological results of the respective tumors (18). With respect to the reference standard domain, 14 studies (19–21, 27–30, 32–38) were regarded as “unknown” because the blinded status of the elastosonography results was not definitely depicted. Regarding the flow and timing domain, 14 studies were regarded as “unknown” because the authors did not definitely mention the precise duration between the reference standard and the elastosonography examination (18–21, 27–33, 36–38).

With regard to applicability, one study was regarded as “high” for the patient selection domain because 12 benign tumors were all pleomorphic adenomas (19). For the index test and reference standard domains, all studies were thought to have low concerns.

### Meta-analysis

**Figure 3:**
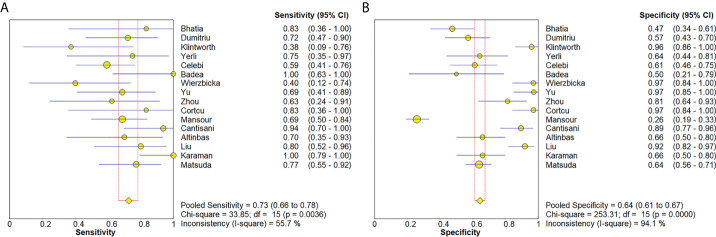
Forest plots for sensitivity **(A)** and specificity **(B)** of elastosonography for diagnosis of malignant salivary gland tumors.

**Figure 4:**
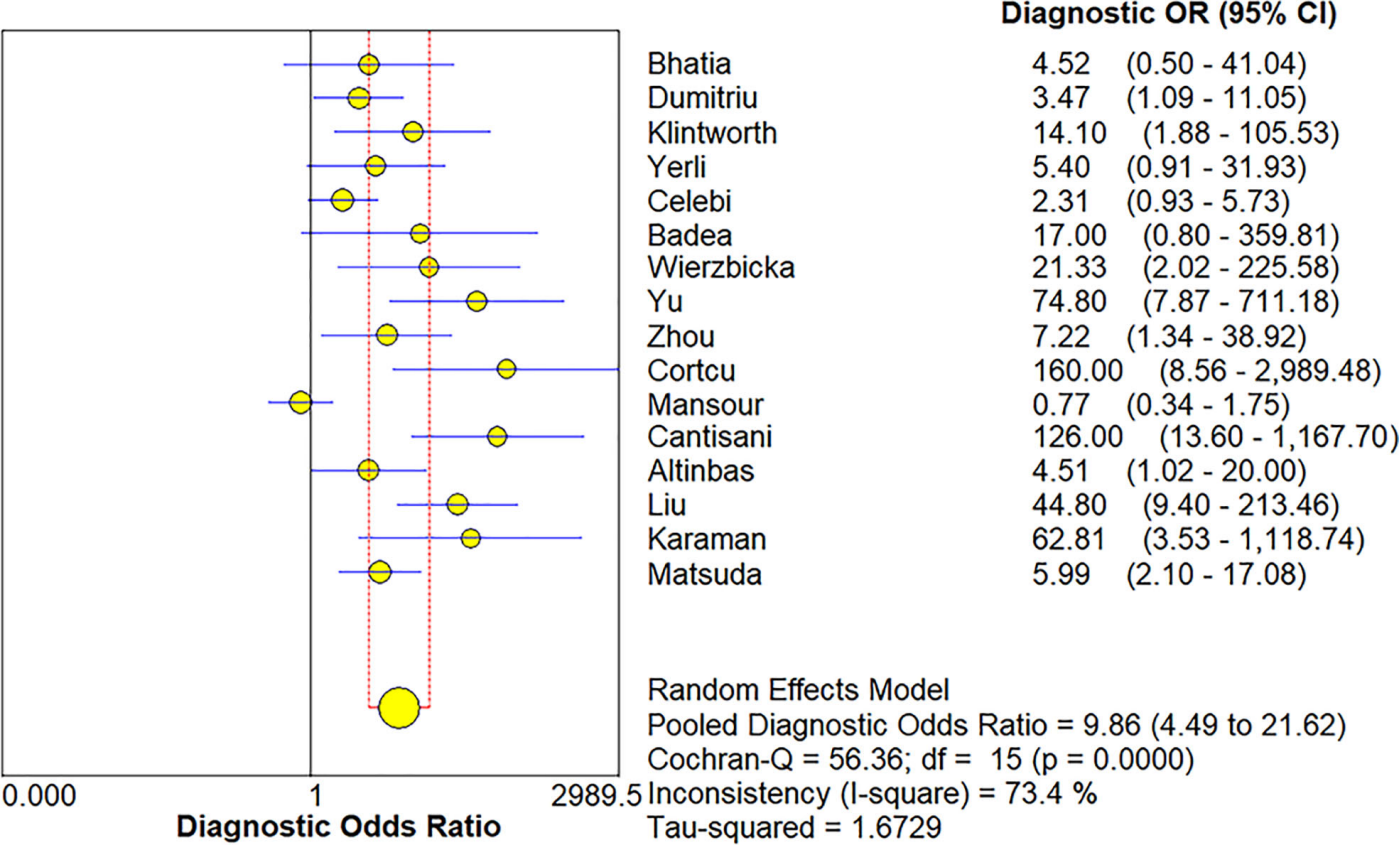
Forest plot for diagnostic odds ratio of elastosonography for diagnosis of malignant salivary gland tumors.

**Figure 5:**
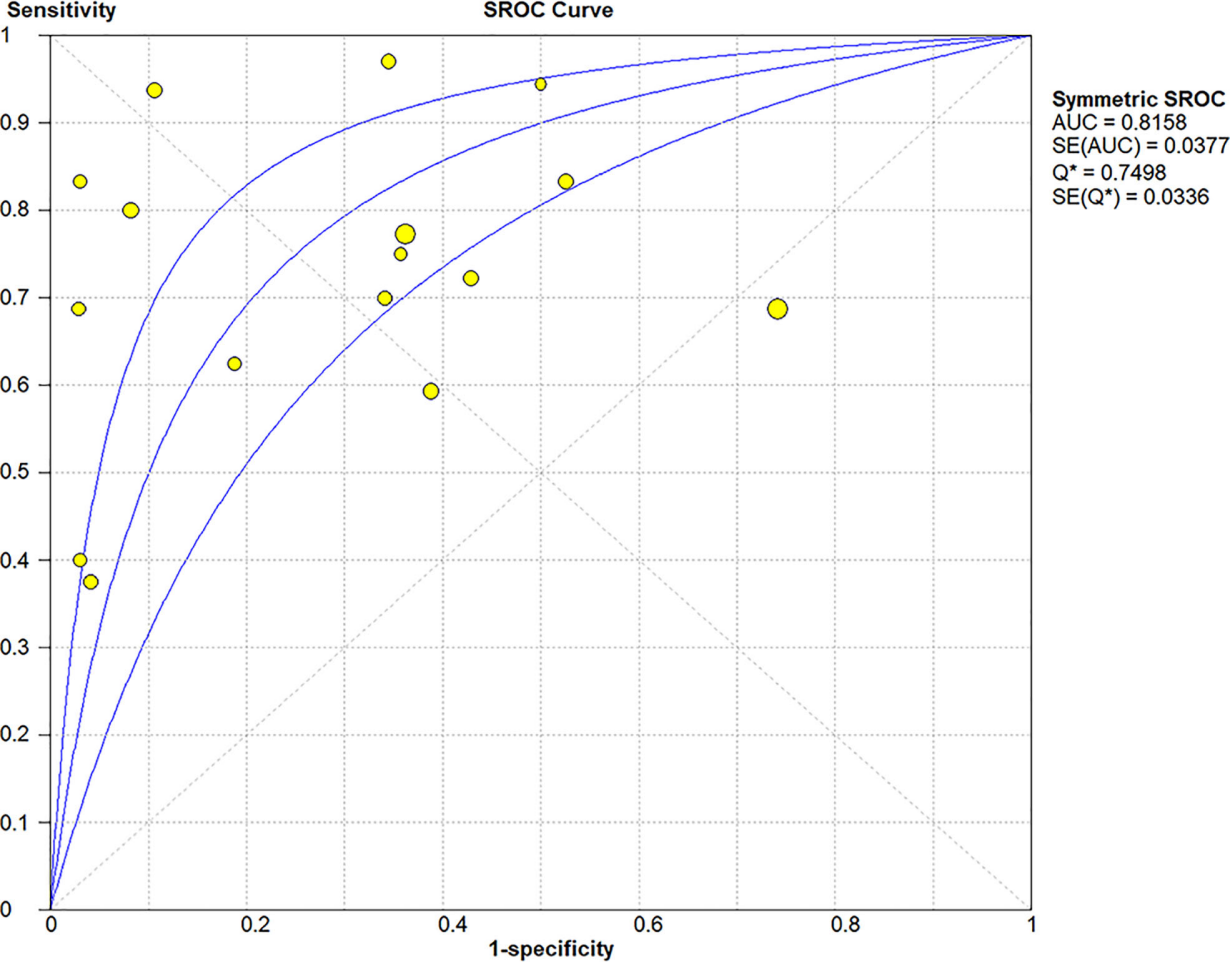
Summary receiver operating characteristic (SROC) curve of elastosonography for diagnosis of malignant salivary gland tumors.

The Spearman correlation coefficient was 0.24 (p = 0.37), indicating that no threshold effect existed. The sensitivities of the 16 enrolled studies ranged from 38.0% to 100.0%, and the specificities ranged from 47.0% to 97.0%. Overall, the pooled sensitivity and specificity of elastosonography for the differentiation between benign and malignant salivary gland tumors were 0.73 (95% CI, 0.66–0.78) and 0.64 (95% CI, 0.61–0.67) (**Figure 3**). The summary estimates of the diagnostic sensitivity and specificity of elastosonography for differentiating benign from malignant salivary gland tumors were analyzed by the random effects method based on significant statistical heterogeneity (*I* ^2^ = 55.7% for sensitivity, p = 0.00; *I* ^2^ = 94.1% for specificity, p = 0.00). The pooled PLR, NLR, and DOR of elastosonography for the differentiation between benign and malignant salivary gland tumors were 2.83 (95%CI, 1.97–4.07), 0.45 (95%CI, 0.32–0.62), and 9.86 (95%CI, 4.49–21.62) (**Figure 4**), respectively. As illustrated in **Figure 5**, the AUC under the SROC curve for the value of elastosonography in the diagnosis of malignant salivary gland tumors was 0.82.

### Meta-regression and subgroup analyses

As a result of the significant heterogeneity among the 16 included studies, a meta-regression analysis was performed to explore potential sources of heterogeneity. The covariates included the blinding method (yes vs. unclear), year of publication (2010**–**2013 vs. 2014**–**2020), study design (prospective vs. others), and assessment methods (quantitative or semiquantitative vs. qualitative). Among the various potential covariates, the assessment methods were associated with the significant heterogeneity (**Table 3**).

**Table 3:** Meta-regression and subgroup analyses.

Covariate	Number of studies	Pooled sensitivity (95% CI)	Pooled specificity (95% CI)	Pooled DOR (95% CI)	AUC	p-Value
**Study design**						0.57
Prospective	10	0.75 (0.67–0.81)	0.61 (0.57–0.65)	12.14 (3.60–40.92)	0.85	
Others	6	0.69 (0.58–0.79)	0.69 (0.64–0.73)	7.20 (3.50–14.81)	0.78	
**Year of publication**						0.23
2010-2013	7	0.64 (0.54–0.74)	0.67 (0.62–0.73)	4.16 (2.32–7.40)	0.72	
2014-2020	9	0.78 (0.70–0.85)	0.63 (0.59–0.67)	16.25 (4.28–61.68)	0.86	
**Assessment method**						0.0034^*^
Quantitative or semiquantitative	4	0.81 (0.68–0.91)	0.93 (0.88–0.96)	73.49 (25.99–207.76)	0.96	
Qualitative	12	0.70 (0.63–0.77)	0.58 (0.54–0.61)	4.72 (2.43–9.17)	0.74	
**Blinding method**						0.24
Yes	12	0.74 (0.67–0.81)	0.73 (0.57–0.65)	12.43 (5.54–27.90)	0.84	
Unclear	4	0.68 (0.55–0.80)	0.43 (0.37–0.49)	4.35 (0.79–23.94)	0.73	

*, Statistical significance (p < 0.05); CI, confidence interval; DOR, diagnostic odds ratio; AUC, area under the curve.

**Figure 6:**
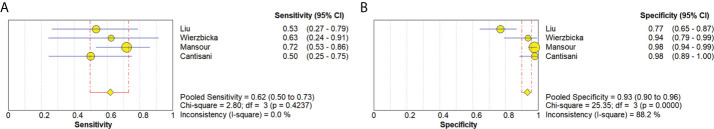
Forest plots for sensitivity **(A)** and specificity **(B)** of conventional ultrasound for diagnosis of malignant salivary gland tumors.

**Figure 7:**
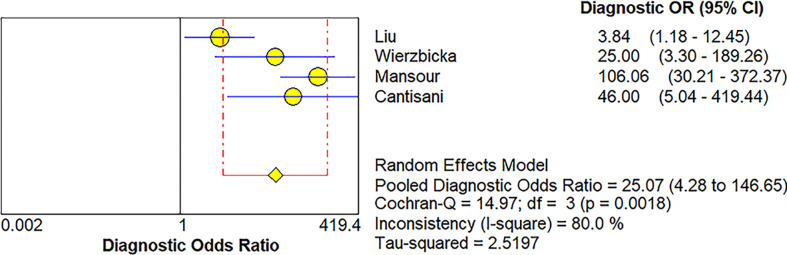
Forest plot for diagnostic odds ratio of conventional ultrasound for diagnosis of malignant salivary gland tumors.

**Figure 8:**
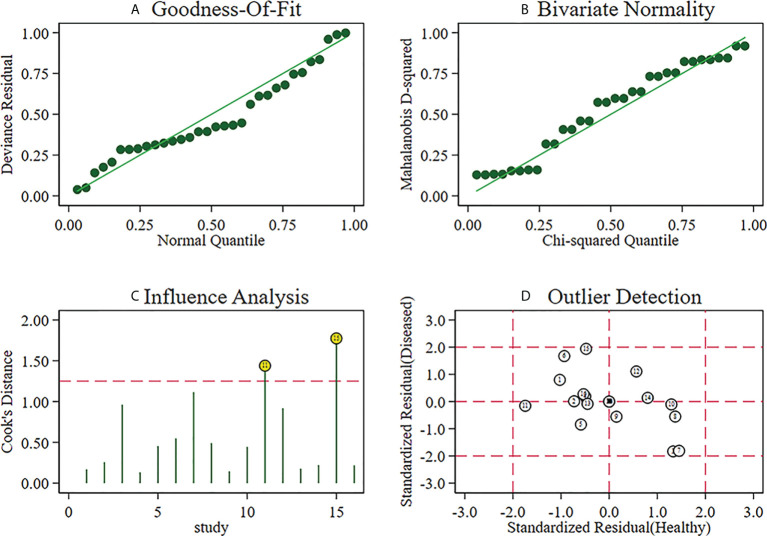
Sensitivity analysis of studies.

Four studies (20, 21, 34, 36) provided data regarding the conventional ultrasound for the differentiation between benign and malignant salivary gland tumors. The pooled sensitivity and specificity of conventional ultrasound for malignant salivary gland tumors were 0.62 (95% CI, 0.50–0.73) and 0.93 (95% CI, 0.90–0.96) (**Figure 6**). The pooled DOR of conventional ultrasound was 25.07 (95%CI, 4.28–146.65) (**Figure 7**). As illustrated in **Figure 8**, the AUC under the SROC curve for the value of conventional ultrasound in the diagnosis of malignant salivary gland tumors was 0.74.

### Sensitivity analysis

A sensitivity analysis was carried out, and the results of the sensitivity analysis found that the meta-analysis results are robust (**Figure 8**).

### Fagan plot analysis and likelihood matrix

**Figure 9:**
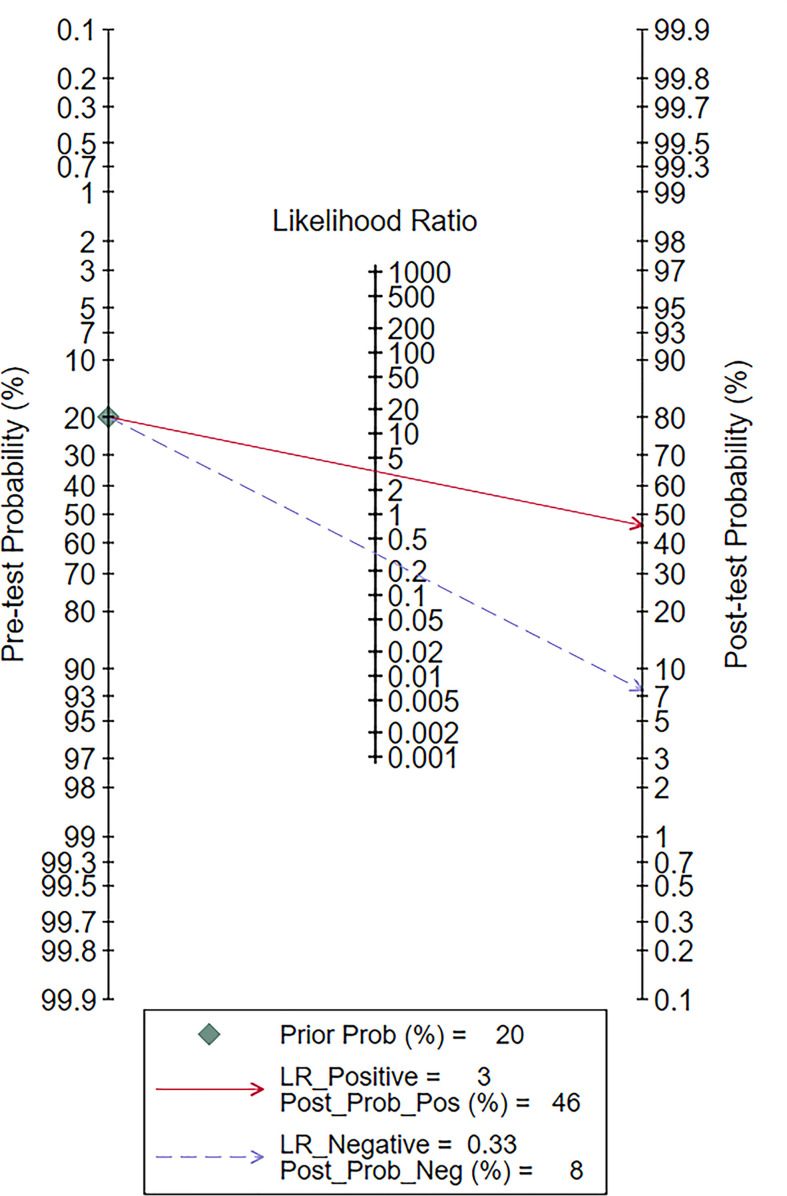
Fagan plot of elastosonography by patient analysis for the diagnosis of malignant salivary gland tumors.

**Figure 10:**
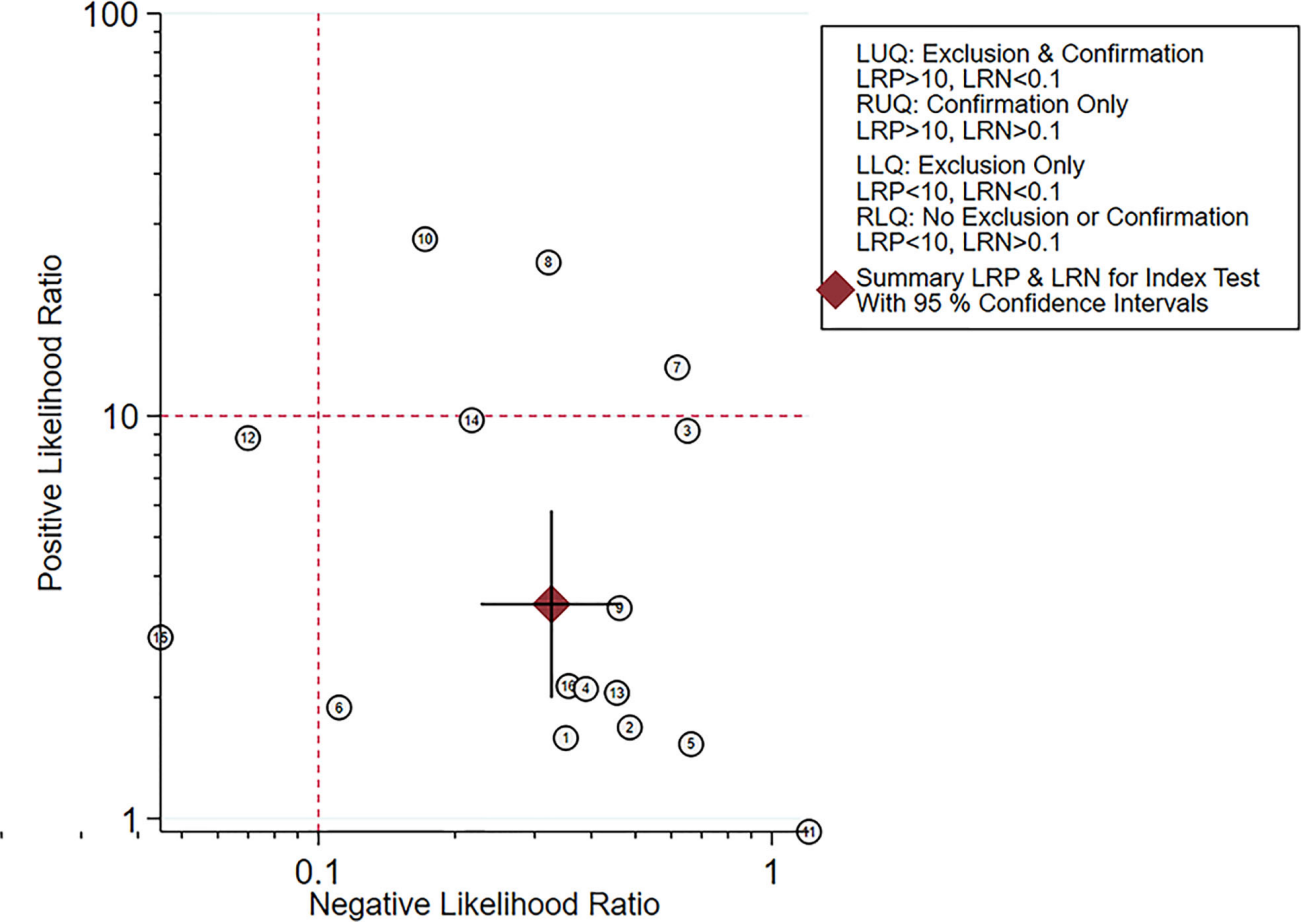
Likelihood matrix indicated that summary positive likelihood ratio and negative likelihood ratio for elastosonography in the diagnosis of malignant salivary gland tumors with 95% confidence intervals were concentrated on the right lower quadrant.

The Fagan diagram was developed for the assessment of clinical application as revealed in **Figure 10**, indicating that when the pretest probability was 20%, the posttest probability was 46% if the results were positive and 8% if the results were negative for malignant salivary gland tumors (**Figure 9**).

The likelihood matrix demonstrated that the summary PLR and NLR for the elastosonography diagnosis of malignant salivary gland tumors with 95% confidence intervals were concentrated on the right lower quadrant, indicating that elastosonography was not effective for malignant salivary gland tumor confirmation and exclusion (**Figure 10**). Therefore, elastosonography is a limited value in the diagnosis of malignant salivary gland tumors.

### Publication bias

**Figure 11:**
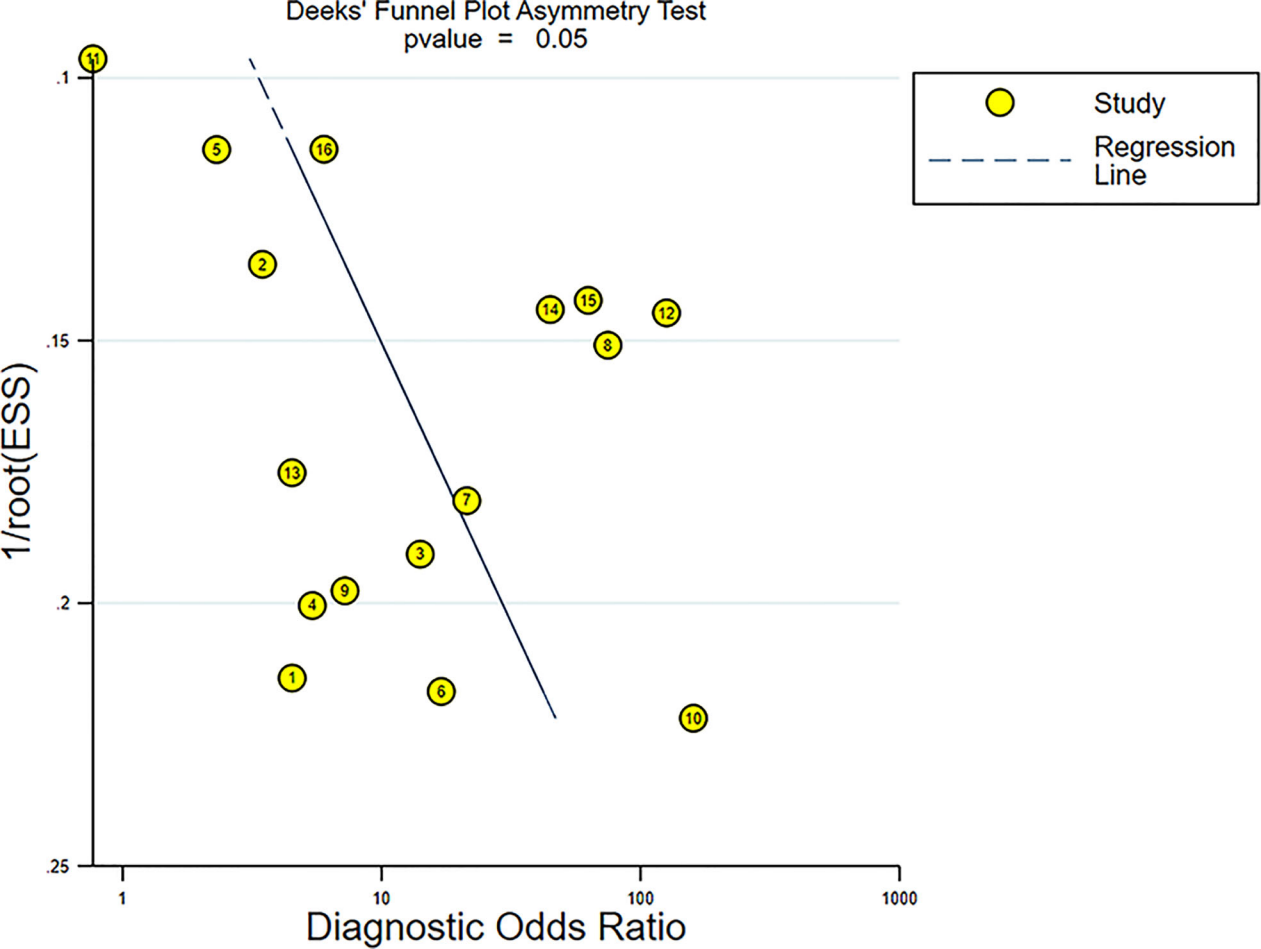
Funnel plot for evaluating potential publication bias.

The Deeks’ funnel plot revealed symmetry in scattered points, suggesting that there was no significant publication bias (p = 0.05) (**Figure 11**).

## Discussion

Our current study found that elastosonography had a pooled moderate sensitivity of 0.73 (95% CI, 0.66–0.78) and a relatively low specificity of 0.64 (95%CI, 0.61–0.67) for the differentiation between benign and malignant salivary gland tumors. The pooled PLR and NLR were 2.83 (95%CI, 1.97–4.07) and 0.45 (95%CI, 0.32–0.62), demonstrating that elastosonography was not effective for malignant salivary gland tumor confirmation and exclusion. The diagnostic odds ratio was 9.86 (95%CI, 4.49–21.62), with an AUC of 0.82. The results indicated that elastosonography revealed a limited value for diagnosing malignant salivary gland tumors.

Four papers supplied the data with regard to conventional ultrasound for the differentiation between benign and malignant salivary gland tumors. The pooled sensitivity, specificity, DOR, and AUC of conventional ultrasound diagnosing malignant salivary gland tumors were 0.62 (95% CI, 0.50–0.73), 0.93 (95% CI, 0.90–0.96), 25.07 (95%CI, 4.28–146.65), and 0.57, respectively. Compared with elastosonography in the diagnosis of salivary gland tumors indirectly, conventional ultrasound had higher specificity (0.93 vs. 0.64), but lower sensitivity (0.62 vs. 0.73), which meant that conventional ultrasound was more effective in the diagnosis of benign salivary gland tumors than of malignant tumors; in contrast, compared with conventional ultrasound, elastosonography had slightly high sensitivity so that it was more effective in the diagnosis of malignant tumors. Consequently, taking the place of utilizing elastosonography or conventional ultrasound alone, the combined use of the two techniques might result in better diagnostic performance. Hence, we believed that elastosonography could be considered as a supplementary diagnostic technique to conventional ultrasound for the assessment of salivary gland tumors.

A prior meta-analysis by Zhang et al. in 2018 (39) included 10 eligible studies on elastosonography for differential diagnosis between benign and malignant parotid lesions, with a total of 725 parotid lesions, and demonstrated that sonoelastography had a limited value for diagnosing malignant parotid lesions with a pooled sensitivity and specificity of 0.67 and 0.64, respectively. Compared with their study, our meta-analysis found that elastosonography had comparable sensitivity (0.73 vs. 0.67) and equal specificity (0.64 vs. 0.64), which confirmed the value of elastosonography in the diagnosis of salivary gland tumors. Furthermore, our study included patients not only with parotid lesions but also with submandibular or sublingual lesions, while Zhang et al.’s study only included patients with parotid lesions. In addition, our meta-analysis enrolled more eligible studies (16 vs. 10 papers). Therefore, we believed that the conclusion of our study might be more generalized.

Another prior meta-analysis by Li et al. (40) included nine eligible articles with 581 tumors using real-time elastography to differentiate benign and malignant salivary gland tumors, and demonstrated moderate diagnostic performance that the pooled sensitivity, specificity, and AUC were 0.76, 0.73, and 0.81, respectively. All the eligible studies adopted strain elastography technology, the traditional form of elastography, which depends on the sonographer’s experience and external manual pressure and is a non-quantitative technology, to assess the stiffness of tumors. In contrast, the included studies in our meta-analysis used not only strain elastography but also shear wave elastography (31, 36), which allows an objective and quantitative assessment of the tumor stiffness (41). The pooled sensitivity and specificity of our meta-analysis were lower compared with Li et al.’s study, whereas our meta-analysis enrolled more recently published articles that not only enhanced the statistical power of this study but also further supported clinical application of elastosonography for diagnosing malignant salivary tumors.

A study by Dumitriu et al. (28) indicated that the depth of the tumor might be a hindrance for elastosonography, which was particularly true for tumors located in the deep parotid lobe. Yerli and colleagues (29) revealed that the assessment of tumors located in the deep parotid lobe was a limitation of conventional ultrasound and was also a limitation of elastosonography. For tumors located in the superficial parotid lobe but relatively deep, the mandibula can hinder the performance of optimal compression in the transverse plane. Furthermore, the mandibula can also affect the performance of optimal longitudinal compression of the submandibular gland. Matsuda et al. (38) found that the sensitivity for differentiating malignant tumors in the superficial parotid lobe was 100%, while the sensitivity was 20% for tumors in the deep lobe, which was attributed to the inability of attenuated acoustic pulses to reach the deep parotid lobe. Therefore, for certain anatomic structures, such as the mandible, the depth of the tumor location and tumors located in different salivary glands might have an effect on the results of elastosonography. However, we were not able to implement meaningful subgroups based on the factors mentioned above, as the data were not recorded in most of the studies.

Although malignant tumors are generally stiffer than benign lesions, a substantial overlap of elastic properties between malignant and benign salivary gland tumors was found in published papers (27, 28, 34). Pleomorphic adenoma, the most common benign salivary gland tumor, is a histologically diverse group of tumors (42), which results in the extremely wide range of elastographic values. In addition, some types of tumors, like Warthin tumors, have variable proportions of solid and cystic components, which would result in a considerable variance in stiffness. Moreover, some benign lesions, inflammatory diseases, as well as abscesses are considered as malignant tumors due to their appearance on elastosonography. It is still significantly difficult to discriminate between benign and malignant salivary gland tumors, and the diagnostic performance of elastosonography is unsatisfying (20, 29, 30). Therefore, other imaging methods complementing elastosonography, such as conventional ultrasound, magnetic resonance imaging, and computed tomography, are needed.

High heterogeneity among the included studies was a major problem in this meta-analysis. The Spearman correlation coefficient was 0.24 (p = 0.37), indicating that no threshold effect existed. Further meta-regression and subgroup analyses revealed that the assessment methods (quantitative or semiquantitative vs. qualitative) might play an important role in the heterogeneity. Quantitative or semiquantitative elastosonography, with higher pooled sensitivity (0.81 vs. 0.70), specificity (0.93 vs. 0.58), DOR (73.49 vs. 4.72), and AUC (0.96 vs. 0.74), performed better than the qualitative one, as shown in **Table 3**. The probable explanation was that compared with qualitative elastosonography, quantitative or semiquantitative elastosonography adopted an algorithm automatically calculated by an ultrasound equipment and was thus less operator-dependent and more objective. Although meta-regression and subgroup analyses excluded the influence of study design, year of publication, and blinding method, other factors such as ultrasound equipment, threshold values, index of elastography, and demographic characteristics would like to be taken into account. Due to the limited included studies, we were not able to perform meaningful subgroups on the basis of other factors mentioned above.

This meta-analysis has some limitations, which should be taken into account while interpreting the conclusions. First, a strict procedure was performed to review the articles and ultimately 16 eligible studies that fulfilled the inclusion criteria were enrolled. There are still relatively rare published studies exploring the value of elastosonography for diagnosis of salivary gland tumors, as the clinical application of elastosonography in the diagnosis of malignant salivary gland tumors was not reported until 2010 (27). Furthermore, only studies written in English were included in our meta-analysis, and then, language bias was inevitable. Second, the comparison between elastosonography and conventional ultrasound was performed indirectly. To determine which imaging modality is superior, a more rigorous research should be carried out adopting these two ultrasound technologies on the same cohort of patients. Finally, methodological limitations in the majority of the included studies were identified, especially in domains including patient selection, index test, reference standard, and flow and timing. Hence, more rigorous studies in the future are needed to address these methodological limitations.

## Conclusions

The existing evidence indicated that elastosonography showed a limited value for diagnosing malignant salivary gland tumors; it could be considered as a supplementary diagnostic technology to conventional ultrasound, and quantitative or semiquantitative elastosonography performed better than the qualitative one. However, large prospective multicenter studies are still needed to validate the conclusion and to further develop the clinical application of elastosonography in salivary gland tumors.

## Data availability statement

The original contributions presented in the study are included in the article/**Supplementary Material**. Further inquiries can be directed to the corresponding author.

## Author contributions

JW, ZW, and GJ designed this study. JW, ZZ, and YJ acquired and analyzed the data. JW, ZZ and XW wrote and edited the manuscript. All authors contributed to the article and approved the submitted version.

## Funding

This project received support from Jinhua Science and Technology Bureau Scientific Research Project (2022-3-019).

## Conflict of interest

The authors declare that the research was conducted in the absence of any commercial or financial relationships that could be construed as a potential conflict of interest.

## Publisher’s note

All claims expressed in this article are solely those of the authors and do not necessarily represent those of their affiliated organizations, or those of the publisher, the editors and the reviewers. Any product that may be evaluated in this article, or claim that may be made by its manufacturer, is not guaranteed or endorsed by the publisher.

## Abbreviations

PA, pleomorphic adenoma; WT, Warthin tumor; US, ultrasound; MRI, magnetic resonance imaging; CT, computed tomography; FNAC, fine-needle aspiration cytology; CNB, core-needle biopsy; MeSH, medical subject heading; PLR, positive likelihood ratio; NLR, negative likelihood ratio; DOR, diagnostic odds ratio; CIs, confidence intervals; SROC, summary receiver operator curve; AUC, area under the curve; *I* ^2^, inconsistency index; SE, strain elasticity; SWE, shear wave velocity; ARFI, acoustic radiation force impulse.
