# Supplementary material for: Diagnostic performance of elastosonography in the differential diagnosis of benign and malignant salivary gland tumors: A meta-analysis
Source: Front Oncol. 2022 Sep 20;12:954751. doi: 10.3389/fonc.2022.954751 (PMC9533713; doi:10.3389/fonc.2022.954751)
Supplement: Supplementary file 1 [file Table_1.doc]

| **Database** | **Search strategy** | **Number** |
| --- | --- | --- |
| Pubmed | (((((Elasticity Imaging Techniques[MeSH Terms]) OR (elasticity ultrasound[Title/Abstract])) OR (ultrasonic elasticity[Title/Abstract])) OR (elasticity ultrasonography[Title/Abstract])) OR (elastosonography[Title/Abstract])) AND ((((((Salivary Gland Neoplasm[MeSH Terms]) OR (Parotid Neoplasm[MeSH Terms])) OR (Sublingual Gland Neoplasm[MeSH Terms])) OR (Submandibular Gland Neoplasm[MeSH Terms])) OR (salivary[Title/Abstract])) OR (parotid[Title/Abstract])) | 95 |
| Embase | #1 Elas*:ab,ti OR sonoelas*:ab,ti  #2 parotid:ab,ti OR salivary:ab,ti OR Submandibular:ab,ti OR Sublingual:ab,ti  #3 #1 AND #2 | 88 |
| Cochrane | #1 (Elas*):ti,ab,kw OR (sonoelas*):ti,ab,kw  #2 (parotid):ti,ab,kw OR (salivary):ti,ab,kw OR Submandibular):ti,ab,kw OR (Sublingua):ti,ab,kw  #3 #1 AND #2 | 27 |

Search strategy
