# Supplementary material for: Diagnostic performance of elastosonography in the differential diagnosis of benign and malignant salivary gland tumors: A meta-analysis
Source: Front Oncol. 2022 Sep 20;12:954751. doi: 10.3389/fonc.2022.954751 (PMC9533713; doi:10.3389/fonc.2022.954751)
Supplement: Supplementary file 2 [file Table_2.doc]

| **Author** | **Type of lesions** |
| --- | --- |
| Bhatia et al. () | Benign nodules: 29 Warthin tumours, 23 pleomorphic adenomas, 3 Kuttner tumours and 4 Kimura’s diseases. Malignant nodules: 2 adenoid cystic carcinomas, 1 adenosquamous carcinoma, 1 metastasis and 2 lymphoma-T cells. |
| Dumitriu et al. () | Benign nodules: 36 Warthin tumours, 13 pleomorphic adenomas, 1 myoepithelioma, 1 oncocytoma, 1 lipoma, 1 neurofibroma, 1 benign lymphoepithelial lesion and 2 epidermoid cysts. Malignant nodules: 5 mucoepidermoid carcinomas, 3 squamous cell carcinomas, 2 adenocarcinomas, 2 adenoid cystic carcinomas, 2 adenosquamous carcinomas, 1 carcinosarcoma and 3 secondary tumors. |
| Klintworth et al. () | Benign nodules: 22 pleomorphic adenomas, 20 Warthin tumors, 2 cystadenomas, 2 lipomas, and 3 cysts. Malignant nodules: 3 squamous cell carcinomas, 1 mucoepidermoid carcinoma, 1 salivary duct carcinoma, 1 basal cell carcinoma, 1 carcinoma ex pleomorphic adenoma, 1 metastatic malignant melanoma. |
| Yerli et al. () | Benign nodules: 11 Warthin tumors, 11 pleomorphic adenomas, 1 lipoma, 3 lymphadenopathies, and 2 abscesses. Malignant nodules: 3 adenocarcinomas, 2 mucoepidermoid carcinomas, 2 adenoid cystic carcinomas, and 1 malignant lymphoma. |
| Celebi et al. () | Benign nodules: 28 pleomorphic adenomas, 10 Warthin tumors, 9 lymphadenopathies, 1 cystic adenoma, and 1 infected cyst rupture. Malignant nodules: 9 lymphomas, 6 mucoepidermoid carcinomas, 5 adenoid cystic carcinomas, 3 metastases, 1 myoepithelial malignant tumor, 2 pleomorphic adenocarcinomas, 2 salivary duct carcinomas, 2 acinic cell carcinomas, and 2 basal cell carcinomas. |
| Badea et al. () | Benign nodules: 12 pleomorphic adenomas. Malignant nodules: 6 parotid carcinomas and 2 metastases. |
| Wierzbicka et al. () | Benign nodules: 23 pleomorphic adenomas, 5 Warthin tumors, 2 monomorphic adenomas, 1 neurofibroma, 1 cyst, and 1 basal cell adenoma. Malignant nodules: 3 adenocarcinomas, 2 lymphomas, 1 clarocellular carcinoma, 1 non-differentiated cancer, 1 squamous cell cancer, 1 cancer from pleomorphic adenoma, and 1 salivary duct cancer. |
| Yu et al. () | Benign nodules: 16 pleomorphic adenomas, 15 adenolymphomas, 2 basal cell adenomas, 1 multiple nodular acidophil adenoma and 1 myoepithelioma. Malignant nodules: 5 mucoepidermoid carcinomas, 3 acinic cell carcinomas, 3 salivary duct carcinomas, 2 basal cell carcinomas, 1 adenoid cystic carcinoma, 1 adenocarcinoma and 1 carcinoma ex pleomorphic adenoma. |
| Zhou et al. () | Benign nodules: 7 Warthin tumors, 17 pleomorphic adenomas, 5 Kimura disease, 1 abscess, 1 branchial cyst, and 1 chronic sialadenitis. Malignant nodules: 1 squamous carcinoma, 2 metastases, 2 mucoepidermoid carcinomas, 1 anaplastic carcinoma, and 2 malignant lymphomas. |
| Cortcu et al. () | Benign nodules: 16 pleomorphic adenomas, 11 warthin tumors, 3 lymph nodes, 2 basal cell adenomas, 1 granulation tissue, and 1 Inflamatuar lesion. Malignant nodules: 1 sarcomatoid carcinoma secondary, 1 epidermoid carcinoma, 1 myxoid liposarcoma metastasis, 1 malignant melanoma metastasis, 1 large cell lymphoma, and 1 low-grade epithelial tumor. |
| Mansour et al. () | Benign nodules: 64 pleomorphic adenomas, 73 Warthin tumors, 6 basal cell adenomas, 3 oncocytomas, 2 polymorphic adenomas, 1 myoepithelioma, 1 sebaceous lymphadenoma, 8 ductal dilatations, 5 lymphoepithelial cysts, 4 cystadenomas, and 3 chronic inflammations. Malignant nodules: 10 primary parotid carcinomas, 13 secondary parotid carcinomas, and 9 non-Hodgkin lymphomas. |
| Cantisani et al. () | Benign nodules: 18 pleomorphic adenomas, 17 Warthin tumors, 5 oncocytomas, 3 lymphoepithelial cysts, 2 ductal cysts, and 2 benign vascular tumors. Malignant nodules: 5 mucoepidermoid carcinomas, 3 malignant lymphomas, 2 squamous cell carcinomas, 2 acinic cell carcinomas, 2 salivary duct carcinomas, and 2 squamous cell carcinoma metastasises. |
| Altinbas et al. () | Benign nodules: 18 Warthin tumors, 8 pleomorphic adenomas, 6 reactive lymphoid hyperplasias, 3 lipomas, 2 granulomatous inflammations, 2 basal cell adenomas, 1 lymphoepithelial cyst, 1 sialadenitis and abscess, 1 sialadenosis, 1 primary benign oncocytic neoplasm, and 1 Rosai-Dorfman disease. Malignant nodules: 3 malignant melanomas, 2 salivary duct carcinomas, 1 squamous cell carcinoma, 1 adenoid cystic carcinoma, 1 Low-grade adenocarcinoma, 1 mucoepidermoid carcinoma, and 1 Merkel cell carcinoma. |
| Liu et al. () | Benign nodules: 26 Warthin tumors, 27 pleomorphic adenomas, 1 myoepithelioma, 1 benign Müllerian tumor, 1 basal cell adenoma, 3 inflammatory nodules, 1 schwannoma, and 1 epidermoid cyst. Malignant nodules: 7 mucoepidermoid carcinomas, 3 malignant mixed Müllerian tumor, 2 acinic cell carcinoma, 2 non–Hodgkin lymphoma, and 1 metastatic melanoma. |
| Karaman et al. () | Benign nodules: 16 lymph nodes, 12 abscesses, 7 pleomorphic adenomas, 5 Warthin tumors, 3 inflammatory changes, and 1 monomorphic adenoma. Malignant nodules: 5 lymph nodes, 6 epithelial tumors, 4 adenoid cystic carcinomas, and 1 recurrent epidermoid cancer. |
| Matsuda et al. () | Benign nodules: 87 Warthin tumors, 68 pleomorphic adenomas, 3 basal cell adenomas, 3 oncocytomas, 1 myoepithelioma and 1 cystadenoma. Malignant nodules: 5 mucoepidermoid carcinomas, 4 salivary duct carcinomas, 4 squamous cell carcinomas, 3 epithelial myoepithelial carcinomas, 2 carcinoma ex pleomorphic adenomas, 2 adenoid cystic carcinomas, 1 myoepithelial carcinoma and 1 large cell undifferentiated carcinoma. |

Histopathological results of the included studies
